# Beneficial effects of maintaining liver function during hepatic arterial infusion chemotherapy combined with tyrosine kinase and programmed cell death protein-1 inhibitors on the outcomes of patients with unresectable hepatocellular carcinoma

**DOI:** 10.1186/s12885-024-12355-x

**Published:** 2024-05-14

**Authors:** Yongqiang Xiao, Wei Deng, Laihui Luo, Guoqing Zhu, Jin Xie, Yu Liu, Renhua Wan, Wu Wen, Zhigao Hu, Renfeng Shan

**Affiliations:** 1 Department of General Surgery, the First Affiliated Hospital, Jiangxi Medical College, Nanchang University, No. 17, Yongwaizheng Street, Nanchang, 330006 Jiangxi China; 2 Department of General Surgery, Ganjiang New Area People’s Hospital, Nanchang, Jiangxi China; 3 Department of General Surgery, Ganjiang New Area Hospital of the First Affiliated Hospital of Nanchang University, Nanchang, Jiangxi China

**Keywords:** Unresectable hepatocellular carcinoma, Hepatic arterial infusion chemotherapy, Programmed cell death protein-1 inhibitors, Tyrosine kinase inhibitors, Hepatic functional reserve

## Abstract

**Background and aim:**

Combination therapy is the primary treatment for unresectable hepatocellular carcinoma (u-HCC). The hepatic functional reserve is also critical in the treatment of HCC. In this study, u-HCC was treated with combined hepatic arterial infusion chemotherapy (HAIC), tyrosine kinase inhibitors (TKIs), and programmed cell death protein-1 (PD-1) inhibitors to analyze the therapeutic response, progression-free survival (PFS), and safety.

**Methods:**

One hundred sixty-two (162) patients with u-HCC were treated by combination therapy of HAIC, TKIs, and PD-1 inhibitors. PFS was assessed by Child–Pugh (CP) classification subgroups and the change in the CP score during treatment.

**Results:**

The median PFS was 11.7 and 5.1 months for patients with CP class A (CPA) and CP class B (CPB), respectively (*p* = 0.013), with respective objective response rates of 61.1 and 27.8% (*p* = 0.002) and conversion rates of 16 and 0% (*p* = 0.078). During treatment, the CP scores in patients with CPA worsened less in those with complete and partial response than in those with stable and progressive disease. In the CP score 5, patients with an unchanged CP score had longer PFS than those with a worsened score (Not reached vs. 7.9 months, *p* = 0.018). CPB was an independent factor negatively affecting treatment response and PFS. Patients with CPA responded better to the combination therapy and had fewer adverse events (AEs) than those with CPB.

**Conclusions:**

Thus, triple therapy is more beneficial in patients with good liver function, and it is crucial to maintain liver function during treatment.

**Supplementary Information:**

The online version contains supplementary material available at 10.1186/s12885-024-12355-x.

## Introduction

Hepatocellular carcinoma (HCC) is a highly prevalent cancer and the second major cause of cancer-related deaths worldwide [1, 2]. Although surgical resection is an effective therapy for HCC, most patients with HCC would have missed the opportunity for curative treatment by the time they are diagnosed [3, 4]. The results of clinical trials such as SHARP [5], REFLECT [6], CheckMate040 [7], and IMbrave150 [8] led to the use of immune checkpoint inhibitors and/or tyrosine kinase inhibitors (TKIs), significantly improving the treatment outcomes in patients with unresectable HCC (u-HCC). This was particularly true for the IMbrave150 trial, in which a combination of medications with various modes of action produced better results than a single-drug treatment approach. Moreover, hepatic arterial infusion chemotherapy (HAIC) showed acceptable tolerability and a comparatively high response rate [9]. Treatments combining the three therapeutic modalities (programmed cell death protein-1 [PD-1] inhibitors, TKIs, and HAIC) might have a synergistic effect in treating u-HCC given their different modes of action, as previously reported [10–13].

It is well known that liver function directly impacts the prognosis of patients with HCC [14]. The most popular liver functional reserve assessment index is the Child–Pugh (CP) classification. The efficacies of HAIC with TKIs and TKIs with PD-1 inhibitors were assessed in patients with HCC based on their liver function [15–18]; however, the efficacy of combined HAIC, TKIs, and PD-1 inhibitors in u-HCC has not been reported.

While overall survival is considered the gold standard for assessing efficacy, the considerable heterogeneity in second-line treatment regimens post-progression and delayed follow-up led to a larger censored group. Therefore, we opted for progression-free survival (PFS) as the primary endpoint in our study [19].

This study categorized patients with u-HCC into CP class A (CPA) and CP class B (CPB) and assessed the therapeutic efficacy and adverse events (AEs) of combined HAIC, TKI, and PD-1 inhibitor therapy. Furthermore, we analyzed factors affecting efficacy and PFS.

## Materials and methods

### Patients

This study included patients with u-HCC treated with a combination of HAIC, TKIs, and PD-1 inhibitors at the First Affiliated Hospital of Nanchang University from October 2020 to April 2022. These patients could not undergo surgery owing to intrahepatic metastases, macrovascular invasion, extrahepatic spread, or insufficient future liver remnants. All patients underwent dynamic computed tomography or magnetic resonance imaging and were diagnosed with HCC following the European Association for the Study of the Liver Clinical Practice Guidelines, Management of Hepatocellular Carcinoma [20].

The ethics committee of the First Affiliated Hospital of Nanchang University approved this study [No. (2022) CDYFYYLK (06–009)] and waived the need for patient informed consent owing to the retrospective nature of this study and as no identifying information was used.

### HAIC

The HAIC procedure was performed as previously described [12, 13, 15]. Briefly, a catheter was inserted from the femoral artery following the Seldinger technique. Based on the arteriography findings, a 2.7 F catheter was inserted into the artery supplying the tumor to deliver the HAIC. The treatment schedules included FOLFOX (HAIC with 5-fluorouracil, oxaliplatin, and leucovorin) and RALOX (HAIC with raltitrexed and oxaliplatin). The chemotherapy drug doses were adjusted according to the patient's CP classification and tolerance to them. The catheter was removed after completing the HAIC procedure and reinserted during the subsequent HAIC cycle.

## TKIs and PD-1 inhibitors

Patients with u-HCC initiated treatment with TKIs and PD-1 inhibitors within three days before or after their initial HAIC session. The included patients were treated with sorafenib, apatinib, or lenvatinib as TKIs and camrelizumab, sintilimab, or tislelizumab as PD-1 inhibitors based on medication availability. Dosage modifications were made following the relevant guidelines, considering the patient's performance, liver function, and treatment tolerance.

## Evaluations

Dynamic computed tomography or magnetic resonance imaging was performed to assess the treatment effectiveness every 4–6 weeks during and after treatment. The modified Response Evaluation Criteria in Solid Tumors (mRECIST) were followed to evaluate treatment effectiveness [21]. The CP score was determined based on a comprehensive physical examination and relevant laboratory test results at each patient visit. PFS was defined as the duration from starting treatment to radiographic progression or death. The disease control rate was calculated as the sum of complete response (CR), partial response (PR), and stable disease (SD) rates. The objective response rate was calculated as the sum of the CR and PR rates.

All treatment-related adverse events (AEs) were identified using the National Cancer Institute Common Terminology Criteria for Adverse Events (Version 5.0). Immune-related AEs were identified, monitored, and tracked following the European Society for Medical Oncology Clinical Practice Guidelines.

## Data collection

The patients' medical records were reviewed, and demographic, clinical, and laboratory data were collected. These included sex, age, Eastern Cooperative Oncology Group performance status (ECOG PS) [22], hepatitis B surface antigen status, alpha-fetoprotein level, Barcelona Clinical Liver Cancer (BCLC) stage [23], macrovascular invasion (portal or hepatic vein tumor thrombosis), number of tumors, maximum tumor diameter, and extrahepatic metastasis status.

## Statistical analysis

The χ^2^ test and Fisher's exact test were performed to evaluate categorical variables. The Student’s *t*-test and Mann–Whitney *U* test were performed to compare continuous variables. The Kaplan–Meier method and the log-rank test were used for PFS analysis. Univariate and multivariable logistic regression analyses were performed to assess treatment effectiveness. A COX proportional hazard model was used for the multivariable examination. Factors with a *p*-value < 0.1 in the univariate analysis were included in the multivariable analysis. Statistical significance was set at *p* < 0.05. Statistical analysis was performed using IBM SPSS Statistics for Windows, Version 26.0 (IBM Corp., Armonk, NY, USA).

## Results

### Patient characteristics

A total of 173 patients were treated with a combination of HAIC, TKIs, and PD-1 inhibitors between October 2020 and April 2022; 11 were lost to follow-up, leaving 162 patients for this research. The patient characteristics are presented in Table 1. The study included 22 females and 140 males. ECOG PS was 0 in 155 patients, 146 were hepatitis B virus (HBV) antigen-positive, and 92 had alpha-fetoprotein levels ≥ 400 ng/mL. Notably, macrovascular invasion and extrahepatic metastases occurred in 75 and 35 patients, respectively. Eight patients with BCLC stage A could not be operated on because of insufficient future liver remnants, and 14 previously received other treatments (transarterial chemoembolization (TACE) alone or with TKIs or PD-1 inhibitors). At the triple therapy initiation, 144 patients were with CPA and 18 with CPB. Among the CPA, 93 patients scored 5, and 51 patients scored 6. All CPB were 7-point patients. The CPA and CPB groups were similar in their clinical characteristics, except for ECOG PS (Table 1).

**Table 1:** Patient demographics and characteristics

Characteristic	All (*n* = 162)	CPA (*n* = 144)	CPB (*n* = 18)	*p*-value
Sex (male/female), *n*	140 /22	125/19	15/3	0.968
Age (< 65/ ≥ 65 years), *n*	132/30	120/24	12/6	0.163
ECOG PS (0/1/2), *n*	155/5/2	141/2/1	14/3/1	0.003
Child–Pugh score (5/6/7)	93/51/18	93/51/0	0/0/18	-
HBV antigen (positive/negative), *n*	146/16	130/14	16/2	0.693
AFP (≥ 400/ < 400 ng/mL), *n*	92/70	85/59	7/11	0.104
Albumin, median [IQR], (g/L)	36.9 (33.6–40.1)	36.9 (33.4–40.1)	38.1 (34.3–40.6)	0.568
Total bilirubin, median [IQR], (µmol/L)	17.4 (11.1–25.1)	17.0 (10.9–24.1)	20.1 (13.5–28.7)	0.205
Number of tumors (1/ > 1), *n*	46/116	41/103	5/13	0.951
Tumor size (≥ 5/ < 5 cm), *n*	145/17	128/16	17/1	0.751
Macrovascular invasion (yes/no), *n*	75/87	64/80	11/7	0.181
Extrahepatic spread (yes/no), *n*	35/127	30/114	5/13	0.710
BCLC stage (A/B/C), *n*	8/44/110	7/41/96	1/3/14	0.532
Previous treatment (yes/no)	14/148	14/130	0/18	0.231

*CPA* Child–Pugh class A, *CPB* Child–Pugh class B, *AFP* alpha-fetoprotein, *ECOG PS* Eastern Cooperative Oncology Group performance status, *HBV* hepatitis B virus, *BCLC* Barcelona Clinic Liver Cancer, *IQR* interquartile range

The predominant treatment regimen for most patients consisted of lenvatinib combined with camrelizumab (*n* = 98), followed by sorafenib combined with camrelizumab (*n* = 20), and lenvatinib combined with sintilimab (*n* = 19) (Supplemental Table S3).

## The effect of treatment

The patients underwent 1–6 HAIC courses (median, 3). Based on mRECIST, 7 (4.3%), 86 (53.1%), 53 (32.7%), and 16 (9.9%) patients, respectively, exhibited CR, PR, SD, and progressive disease (PD), with an objective response rate of 57.4% (Supplemental Table S2). Compared to those of the CPB group, the CPA group had a high objective response rate (61.1 vs. 27.8%, *p* = 0.002) and disease control rate (91 vs. 83.3%, *p* = 0.014; Supplemental Table S2). Interestingly, 23 patients with CPA underwent surgical resection after senior surgeons verified during a multidisciplinary meeting that adequate future liver would remain (Supplemental Table S2).

**Fig. 1:**
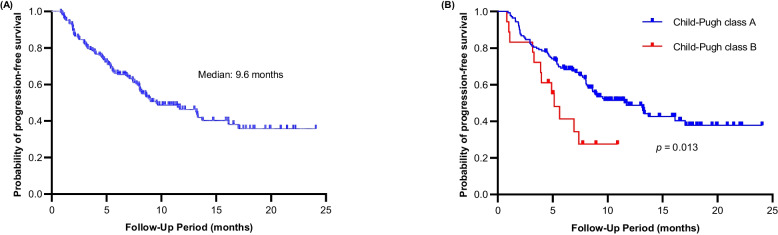
**A** Kaplan–Meier curve analysis of progression-free survival for all patients undergoing the combined HAIC, TKI, and PD-1 inhibitor treatment (triple therapy). **B** Kaplan–Meier curves indicating progression-free survival for patients undergoing the triple therapy and stratified by their Child–Pugh classification

The median PFS for all patients was 9.6 months (Fig. 1A). There were too few cases to establish the median overall survival. Patients with CPA had a longer median PFS than those with CPB (11.7 vs. 5.1 months, *p* = 0.013; Fig. 1B).

## Univariate and multivariable analyses of clinical factors associated with the PFS following combined HAIC, TKI, and PD-1 inhibitor treatment

Univariate analysis of baseline clinical characteristics indicated that CP classification (*p* = 0.015), number of tumors (*p* = 0.084), macrovascular invasion (*p* = 0.084), and extrahepatic spread (*p* = 0.002) were significantly associated with PFS (Table 2). Multivariable analysis with these factors revealed that CP classification [hazard ratio (HR) 2.209; 95% confidence interval (CI), 1.176–4.418; *p* = 0.014], the number of tumors (HR, 1.809; 95% CI, 1.072–3.052; *p* = 0.026), and the Extrahepatic spread (HR, 2.046; 95% CI, 1.244–3.365; *p* = 0.005) were significant independent predictors of PFS (Table 2).

**Table 2:** Univariate and multivariable analyses of factors associated with progression-free survival

	Univariate analysis	Multivariable analysis		
Variable	*p-*value	HR	95% CI	*p-*value
Sex (male/female)	0.596	-	-	-
Age (< 65/ ≥ 65 years)	0.773	-	-	-
Child–Pugh class (A/B)	0.015	2.209	1.176–4.418	0.014
ECOG PS (0/1, 2)	0.133	-	-	-
HBV antigen (negative/positive)	0.766	-	-	-
AFP (< 400/ ≥ 400 ng/mL)	0.186	-	-	-
Number of tumors (1/ > 1)	0.084	1.809	1.072–3.052	0.026
Tumor size (< 5/ ≥ 5 cm)	0.565	-	-	-
Macrovascular invasion (yes/no)	0.084	1.487	0.953–2.318	0.080
Extrahepatic spread (yes/no)	0.002	2.046	1.244–3.365	0.005
Previous treatment (yes/no)	0.220	-	-	-

*AFP* alpha-fetoprotein, *ECOG PS* Eastern Cooperative Oncology Group performance status, *HBV* hepatitis B virus, *HR* hazard ratio, *CI* confidence interval

## Effect of the combined HAIC, TKI, and PD-1 inhibitor therapy on the CP score during the treatment period

**Fig. 2:**
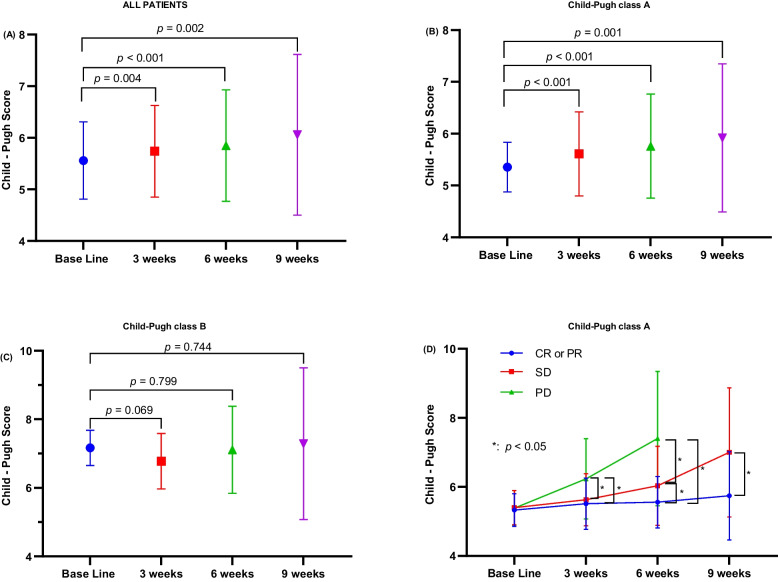
**A** The baseline and weeks 3, 6, and 9 Child–Pugh scores in all patients undergoing the combined HAIC, TKI, and PD-1 inhibitor treatment (triple therapy). **B** The baseline and weeks 3, 6, and 9 Child–Pugh scores in patients with Child–Pugh class A undergoing the triple therapy. **C** The baseline and weeks 3, 6, and 9 Child–Pugh scores in patients with Child–Pugh class B undergoing the triple therapy. **D** The baseline and weeks 3, 6, and 9 Child–Pugh scores in patients with Child–Pugh class A undergoing the triple therapy and stratified by their treatment response. CR, complete response; PR, partial response; SD, stable disease; PD, progressive disease

The CP score information was available for 162, 124, and 70 patients 3, 6, and 9 weeks after initiating the triple therapy. The mean CP score at baseline and 3, 6, and 9 weeks into the treatment were 5.54 ± 0.75, 5.74 ± 0.89, 5.85 ± 1.08, and 6.06 ± 1.56, respectively (Fig. 2A). The CP score for the entire cohort at baseline differed significantly from those at weeks 3, 6, and 9; however, subgroup analysis revealed that these differences were present in the CPA but not the CPB group (Figs. 2B & 2C). Although the CP scores in the CR + PR, SD, and PD subgroups of the CPA group worsened, the CR + PR subgroup had significantly higher CP scores than the SD and PD subgroups (Fig. 2D).

**Fig. 3:**
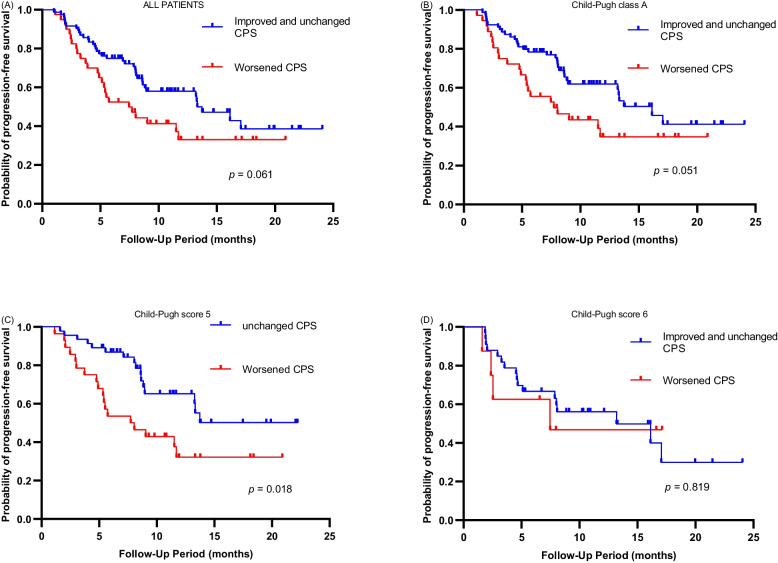
**A** Kaplan–Meier curve for progression-free survival in all patients undergoing the combined HAIC, TKI, and PD-1 inhibitor treatment (triple therapy) according to how their Child–Pugh score changed. **B** Kaplan–Meier curve for progression-free survival in patients with Child–Pugh class A undergoing the triple therapy according to how their Child–Pugh score changed. **C** Kaplan–Meier curve for progression-free survival in patients with Child–Pugh score 5 undergoing the triple therapy according to how their Child–Pugh score changed. **D** Kaplan–Meier curve for progression-free survival in patients with Child–Pugh score 6 undergoing the triple therapy according to how their Child–Pugh score changed. CPS, Child–Pugh score

Additionally, the analysis revealed an insignificantly shorter PFS in patients with worsened CP scores after 6 weeks of treatment than in those with improved or unchanged scores for the entire cohort (13.3 vs. 7.5 months, *p* = 0.061; Fig. 3A) and patients with CPA (16.1 vs. 7.7 months; *p* = 0.051; Fig. 3B). Subgroup analysis of patients with CP scores 5 and 6 in the CPA group showed a better PFS in patients with unchanged CP scores than in those with worsened CP scores in the CP score 5 subgroup (Not reached vs. 7.9 months, *p* = 0.018; Fig. 3C) but not in the CP score 6 subgroup (*p* = 0.819; Fig. 3D).

## Determinants of response to the combination therapy in patients with CPA

Univariate analysis showed that the number of tumors (*p* = 0.027), macrovascular invasion (*p* = 0.080), extrahepatic spread (*p* = 0.071), the BCLC stage (*p* = 0.002), and CP score change after 6 weeks of treatment (unchanged or improved *versus* worsened scores; *p* = 0.058) were associated with treatment response (CR and PR; Supplemental Table S1). Multivariable analysis with these variables showed that the number of tumors (odds ratio [OR], 3.590; 95% CI, 1.191–10.819; *p* = 0.023) and the Extrahepatic spread (OR, 2.888; 95% CI, 1.105–7.546; *p* = 0.030) were significant independent predictors of response to the combined treatment (Table 3).

**Table 3:** Univariate and multivariable analyses for factors associated with treatment response in patients with CPA

	Univariate analysis			Multivariate analysis		
Variable	OR	95% CI	*p*-value	OR	95% CI	*p*-value
Sex (male/female)	0.518	0.176–1.528	0.234	-	-	-
Age (< 65/ ≥ 65 years)	1.149	0.471–2.801	0.760	-	-	-
HBV antigen (yes/no)	1.667	0.496–5.597	0.409	-	-	-
AFP (< 400/ ≥ 400 ng/mL)	1.267	0.638–2.515	0.499	-	-	-
Number of tumors (1/ > 1)	2.502	1.111–5.634	0.027	3.590	1.191–10.819	0.023
Tumor size (< 5/ ≥ 5 cm)	1.068	0.366–3.123	0.904	-	-	-
Macrovascular invasion (yes/no)	1.833	0.930–3.611	0.080	2.267	0.927–5.547	0.073
Extrahepatic spread (yes/no)	2.114	0.937–4.771	0.071	2.888	1.105–7.546	0.030
Child–Pugh score change^a^	2.256	0.974–5.224	0.058	1.838	0.738–4.582	0.191
Previous treatment (no/yes)	2.278	0.746–6.959	0.149	-	-	-

*CPA* Child–Pugh class A, *OR* odds ratio, *AFP* alpha-fetoprotein, *ECOG PS* Eastern Cooperative Oncology Group performance status, *HBV* hepatitis B virus, *CI* confidence interval

^a^Child–Pugh score change after 6 weeks of combined treatment with HAIC, TKIs, and PD-1 inhibitors (improved and unchanged *versus* worsened Child–Pugh score)

## Adverse events

The AEs observed during the combined therapy are presented in Supplemental Table S2. Increased levels of transaminases, fatigue, and nausea and vomiting were the three most prevalent AEs. While some severe grade 3 or 4 AEs were identified, most were of grade 1 or 2, and no fatal AEs was identified. Notably, the incidence of grade 3 and 4 AEs was higher in the CPB group than in the CPA group and included fatigue [2 (1.4%) vs. 3 (16.7%); *p* = 0.015], abdominal pain [2 (1.4%) vs. 5 (27.8%); *p* < 0.001], nausea and vomiting [2 (1.4%) vs. 3 (16.7%); *p* = 0.010], diarrhea [1 (0.7%) vs. 2 (11.1%); *p* = 0.033], reactive cutaneous capillary endothelial proliferation [1 (0.7%) vs. 2 (11.1%); *p* = 0.033], hypertension [1 (0.7%) vs. 2 (11.1%); *p* = 0.033)], and increased levels of transaminases [6 (4.2%) vs. 4 (22.2%); *p* = 0.015; Supplemental Table S3).

## Discussion

In this study, combination therapy of HAIC, TKIs, and PD-1 inhibitors showed better ORR and PFS in patients with CPA than those with CPB. Multivariate analysis revealed that CPB was an independent factor negatively affecting PFS. Additionally, patients with improved or unchanged CP scores during treatment may indicate better PFS and a greater likelihood of subsequent surgical treatment. These results suggest that u-HCC patients with good liver function have better benefits from triple therapy and that maintaining better liver function over the course of treatment is more likely to be treated surgically.

Prior research has demonstrated that triple therapy improved ORR, DCR, and PFS, [10–12] and this information is generally consistent with our findings. Additionally, participants in our research who had improved liver function had better ORR, DCR, PFS, and fewer AEs. These findings might be related to poorer hepatic reserve and impaired metabolism caused by the therapeutic agents in patients with CPB, resulting in higher drug levels in their bodies with a greater susceptibility to AEs, especially more serious ones. Studies have shown that patients with poor hepatic reserve have more AEs and difficulty in maintaining doses, and the administered treatment has a lower antitumor efficacy [24, 25]. Analysis of factors that might affect the patients' PFS suggested that poor CP classification could be an independent risk factor in u-HCC; therefore, patients with poor hepatic functional reserve might be less likely to benefit from the triple therapy than those with good hepatic reserve. HAIC is based on infusing chemotherapeutic agents through the hepatic artery; however, oxaliplatin might cause hepatic sinusoidal injury [26], 5-fluorouracil might induce an inflammatory response [27], and both might increase leukocyte antigen expression and enhance T lymphocyte stimulation, activating the acquired immune system [28]. These actions might cause or exacerbate immune checkpoint inhibitor-mediated liver injury and reactivate hepatitis viruses even though all patients with HBV receive antiviral therapy. Furthermore, PD-1 inhibitors might directly kill hepatocytes through complement-mediated tissue inflammation and induction of cytokine secretion by immune cells [29, 30]; TKIs can directly damage hepatocytes, cause cholestasis and mediate hepatocyte steatosis, all of which can further aggravate liver injury. All these variables considerably restrict using the triple therapy in individuals with impaired liver function. Additionally, our analysis of the 23 surgically treated patients revealed that their pre-treatment CPA classification remained unchanged throughout treatment and before surgery. Maintaining a greater hepatic function could facilitate eventual access to curative treatments. Given that the majority of our cohort had hepatitis B-related hepatocellular carcinoma, antiviral therapy may suppress hepatitis B virus replication, thereby mitigating hepatocellular injury. Additionally, precise artery superselection during HAIC may minimize hepatocyte damage in non-targeted regions, potentially preserving liver function during treatment.

This study revealed that the PFS after 6 weeks of treatment was longer in patients with non-worsening CP scores than in those with worsening CP scores, especially in patients with a good liver function (CP score 5) at the time of treatment. Liver function is typically impacted by intrahepatic lesions and portal vein tumor thrombi. When HAIC is used with TKIs, the tumor burden is drastically reduced, and, in some cases, the macrovascular tumor thrombus is resolved [10]. TKIs cause tumor ischemia owing to their anti-VEGF effect, reducing its burden [31–33]. The tumor cell killing effect of PD-1 inhibitor-mediated cellular immunity further reduces tumor burden [34], and combined treatment with TKIs and PD-1 inhibitors could promote vascular normalization [35]. Moreover, these drugs could improve the hepatic functional reserve. Continued worsening of the hepatic functional reserve during treatment might correlate with increased tumor burden and advancement of the macrovascular carcinoma thrombus, frequently indicating poor therapeutic response and a reduced PFS. In addition, triple therapy may not be continued due to deterioration in liver function, making treatment ineffective; and, other treatments may not be available after progression, although the median OS was not achieved in our study, as reported by Mei et al. [11] showed that OS was significantly better with triple therapy than with TKIs plus PD-1 inhibitors (15.9 months vs. 8.6 months, *p* = 0.0015).and that CPB was an independent negative factor for OS. Furthermore, individuals with HCC are often linked with cirrhosis, which generally indicates worse liver function and weak regenerative capacity of the liver, also accompanied by the expression of drug-resistant genes, which may be associated with poorer treatment effectiveness [36–38].

The univariate analysis demonstrated that changes in the number of tumors, macrovascular invasion, extrahepatic spread, and the CP score during treatment were associated with the patient response to the triple therapy. We further determined potential variables associated with the outcomes in patients with CPA. Our findings are consistent with previous reports [15–18]. Notably, changes in the CP score during treatment impacted the treatment response significantly. Therefore, monitoring the hepatic functional reserve during treatment is critical, even in patients with good baseline hepatic function (CPA).

Patients with u-HCC and CPA treated with combined HAIC, TKIs, and PD-1 inhibitors responded better to treatment, had longer PFS, and had fewer AEs than similar patients with CPB. Furthermore, changes in the CP score during treatment correlated with the response to treatment and PFS and should be closely monitored.

This study has several limitations. First, it was a single-center, retrospective, non-randomized study with no control group, making it vulnerable to various confounding factors. Validation of our findings in prospective, multicenter, randomized, controlled trials is required. Second, the small number of patients in the CPB group may have impacted the results. Further validation in a larger sample is required. Finally, the number of patients for whom overall survival was observed in the current study was too small for analysis. Long-term survival data are still lacking.

## Conclusion

This retrospective study showed that the combined HAIC, TKI, and PD-1 inhibitor therapy was efficient in patients with u-HCC and that patients with CPA had better treatment responses, fewer AEs, and longer PFS than patients with CPB. Furthermore, patients with relatively stable CP scores responded better to treatment than those with fluctuating scores, and those with non-worsening CP scores had longer PFS than those with worsening scores. A longer follow-up and a larger number of cases are needed to examine the parameters correlated to treatment response and persistence and obtain more conclusive results.

### Supplementary Information

Supplementary Material 1.

## Data Availability

The data that support the results of this study are available upon request from the corresponding author.

## Abbreviations

AEs: Adverse events
BCLC: Barcelona Clinical Liver Cancer
CI: Confidence interval
CP: Child–Pugh
CPA: Child–Pugh class A
CPB: Child–Pugh class B
CR: Complete response
ECOG PS: Eastern Cooperative Oncology Group performance status
FOLFOX: HAIC with 5-fluorouracil, oxaliplatin, and leucovorin
HAIC: Hepatic arterial infusion chemotherapy
HBV: Hepatitis B virus
HCC: Hepatocellular carcinoma
HR: Hazard ratio
mRECIST: Modified Response Evaluation Criteria in Solid Tumors
OR: Odds ratio
PD: Progressive disease
PD-1: Programmed cell death protein-1
PFS: Progression-free survival
PR: Partial response
RALOX: HAIC with raltitrexed and oxaliplatin
SD: Stable disease
TACE: Transarterial chemoembolization
TKIs: Tyrosine kinase inhibitors
u-HCC: Unresectable hepatocellular carcinoma
