# Supplementary material for: Beneficial effects of maintaining liver function during hepatic arterial infusion chemotherapy combined with tyrosine kinase and programmed cell death protein-1 inhibitors on the outcomes of patients with unresectable hepatocellular carcinoma
Source: BMC Cancer. 2024 May 14;24:588. doi: 10.1186/s12885-024-12355-x (PMC11092091; doi:10.1186/s12885-024-12355-x)
Supplement: Supplementary file 1 — Supplementary Material 1. [file 12885_2024_12355_MOESM1_ESM.docx]

Supplemental Table S1. Different combinations of TKIs and PD-1 inhibitors

| Drug combinations | Patients, *n* |
| --- | --- |
| Lenvatinib + Camrelizumab | 98 |
| Lenvatinib + Sintilimab | 19 |
| Lenvatinib + Tislelizumab | 10 |
| Apatinib + Camrelizumab | 9 |
| Sorafenib + Camrelizumab | 20 |
| Sorafenib + Sintilimab | 6 |

Supplemental Table S2. Response to HAIC combined with TKIs and PD-1 inhibitors

| Evaluation | All patients  (*n* = 162) | CPA  (*n* = 144) | CPB  (*n* =18) | *p-*value |
| --- | --- | --- | --- | --- |
| Complete Response, *n* (%) | 7 (4.3) | 7 (4.9) | 0 (0) | - |
| Partial Response, *n* (%) | 86 (53.1) | 81 (56.2) | 5 (27.8) | - |
| Stable Disease, *n* (%) | 53(32.7) | 43 (29.9) | 10 (55.5) | - |
| Progressive Disease, *n* (%) | 16(9.9) | 13 (9.0) | 3 (16.7) | - |
| Objective Response Rate (%) | 57.4 | 61.1 | 27.8 | 0.002 |
| Disease Control Rate (%) | 90.1 | 91.0 | 83.3 | 0.014 |
| Conversion rate, *n* (%) | 23 (14.2) | 23 (16.0) | 0 (0) | 0.078 |

CPA, Child–Pugh class A; CPB, Child–Pugh class B.

Supplemental Table S3. Adverse events associated with HAIC combined with TKIs and PD-1 inhibitors treatment

|  | ALL  (*n* = 162) | | CPA  (*n* = 144) | | CPB  (*n* = 18) | | *p*-value | |
| --- | --- | --- | --- | --- | --- | --- | --- | --- |
| Adverse event | any grade,  n (%) | grade 3/4,  n (%) | any grade,  n (%) | grade 3/4, n (%) | any grade, n (%) | grade 3/4,  n (%) | any grade | grade 3/4 |
| Fever | 25(15.4) | 0(0) | 19(13.2) | 0(0) | 6(33.3) | 0(0) | 0.038 | - |
| Fatigue | 97(59.9) | 5(3.1) | 90(62.5) | 2(1.4) | 7(38.9) | 3(16.7) | 0.074 | 0.015 |
| weight loss | 43(26.5) | 0(0) | 39(27.1) | 0(0) | 4(22.2) | 0(0) | 0.783 | - |
| Abdominal pain | 57(35.2) | 7(4.3) | 49(34.0) | 2(1.4) | 8(44.4) | 5(27.8) | 0.436 | <0.001 |
| Nausea and Vomiting | 71(43.8) | 5(3.1) | 64(44.4) | 2(1.4) | 7(38.9) | 3(16.7) | 0.802 | 0.010 |
| Diarrhea | 15(9.3) | 3(1.9) | 10(6.9) | 1(0.7) | 5(27.8) | 2(11.1) | 0.014 | 0.033 |
| Rash | 16(9.9) | 2(1.2) | 12(8.3) | 1(0.7) | 4(22.2) | 1(5.6) | 0.083 | 0.211 |
| Hand-foot skin reaction | 53(32.7) | 7(4.3) | 48(33.3) | 5(3.5) | 5(27.8) | 2(11.1) | 0.792 | 0.175 |
| RCCEP | 37(22.8) | 3(1.9) | 31(21.5) | 1(0.7) | 6(33.3) | 2(11.1) | 0.250 | 0.033 |
| Hypertension | 29(17.9) | 3(1.9) | 22(15.3) | 1(0.7) | 7(38.9) | 2(11.1) | 0.022 | 0.033 |
| Hypothyroid-ism | 12(7.4) | 2(1.2) | 9(6.3) | 1(0.7) | 2(11.1) | 1(5.6) | 0.351 | 0.211 |
| Decreased appetite | 54(33.3) | 0(0） | 47(32.6) | 0(0) | 7(38.9) | 0(0) | 0.604 | - |
| Ascites | 34(21.0) | 4(2.5) | 26(18.1) | 2(1.4) | 8(44.4) | 2(11.1) | 0.026 | 0.061 |
| Pleural effusion | 19(11.7) | 1(0.6) | 16(11.1) | 0(0) | 3(16.7) | 1(5.6) | 0.447 | 0.112 |
| Myocarditis | 2(1.2) | 1(0.6) | 1(0.7) | 0(0) | 1(5.6) | 1(5.6) | 0.211 | 0.112 |
| Increased blood bilirubin | 61(37.7) | 9(5.6) | 55(38.2) | 6(4.2) | 6(33.3) | 3(16.7) | 0.800 | 0.064 |
| Increased transaminase | 130(80.2) | 10(6.2) | 118(81.9) | 6(4.2) | 12(66.7) | 4(22.2) | 0.204 | 0.015 |
| Decreased Platelet count | 35(21.6) | 8(4.9) | 30(20.8 | 6(4.2) | 5(27.8) | 2(11.1) | 0.545 | 0.218 |
| Proteinuria | 8(4.9) | 0(0) | 6(4.2) | 0(0) | 2(11.1) | 0(0) | 0.218 | - |

CPA, Child–Pugh class A; CPB, Child–Pugh class B. RCCEP: Reactive cutaneous capillary endothelial proliferation
